# Engineering psychrophilic polymerase for nanopore long-read sequencing

**DOI:** 10.3389/fbioe.2024.1406722

**Published:** 2024-07-01

**Authors:** Yaping Sun, Danny Hsu Ko, Jie Gao, Kang Fu, Yuanchen Mao, Yun He, Hui Tian

**Affiliations:** Research Center of Molecular Diagnostics and Sequencing, Research Institute of Tsinghua University in Shenzhen, Shenzhen, China

**Keywords:** psychrophilic polymerases, incorporation of polymer-tagged nucleotides, salt tolerance, exonuclease activity, sequencing by synthesis (SBS)

## Abstract

Unveiling the potential application of psychrophilic polymerases as candidates for polymerase-nanopore long-read sequencing presents a departure from conventional choices such as thermophilic *Bacillus* stearothermophilus (Bst) renowned for its limitation in temperature and mesophilic *Bacillus subtilis* phage (phi29) polymerases for limitations in strong exonuclease activity and weak salt tolerance. Exploiting the PB-Bst fusion DNA polymerases from Psychrobacillus (PB) and *Bacillus* stearothermophilus (Bst), our structural and biochemical analysis reveal a remarkable enhancement in salt tolerance and a concurrent reduction in exonuclease activity, achieved through targeted substitution of a pivotal functional domain. The sulfolobus 7-kDa protein (Sso7d) emerges as a standout fusion domain, imparting significant improvements in PB-Bst processivity. Notably, this study elucidates additional functional sites regulating exonuclease activity (Asp43 and Glu45) and processivity using artificial nucleotides (Glu266, Gln283, Leu334, Glu335, Ser426, and Asp430). By disclosing the intricate dynamics in exonuclease activity, strand displacement, and artificial nucleotide-based processivity at specific functional sites, our findings not only advance the fundamental understanding of psychrophilic polymerases but also provide novel insights into polymerase engineering.

## 1 Introduction

Long-read sequencing approach extracts whole-genome information concerning complex structural variations more effectively compared to short-read sequencing systems with read length smaller than 300 bp and enables broader applications in clinical diagnostics of genetic diseases (Byrne et al., 2019; Sakamoto et al., 2020; Tedersoo et al., 2021). The polymerase-nanopore complex assumes a pivotal role in long-read sequencing systems requiring polymerases with robust strand-displacement activity, enhanced salt tolerance, which is essential in achieving high signal-to-noise ratio, and efficient incorporation of modified substrates. The strand-displacement activity of polymerases enables consensus reading of circular templates, a strategy to increase sequencing accuracy. On the other hand, modified substrates instead of natural deoxyribonucleotide triphosphates (dNTPs) are often required to generate detectable signals, thus compatibility to such substrates determines the polymerase-nanopore sequencing read length. Exonuclease activity of polymerases is less desirable because it leads to the degradation of oligonucleotide-modified substrates and primed templates (Fuller et al., 2016).

Phi29 polymerase from the *Bacillus subtilis* phage phi29 is widely used in nanopore sequencing systems due to its strong strand displacement activity and processivity at 30°C (Stranges et al., 2016). In this electrochemical sequencing systems, high salt concentrations, such as 300 mM NaCl buffer, are required to reduce the overall solution resistance of the electrochemical cell, thereby improving the signal-to-noise ratio and enabling the quantification of small changes in current or potential (Davis and Chen, 2013; Stranges et al., 2016; Bard et al., 2022; Sun et al., 2023). However, the processivity of phi29 polymerase is greatly compromised under high-salt conditions due to higher tendency of dissociation from DNA templates (Sun et al., 2023). Fusing DNA binding modules such as helix–hairpin–helix (HhH)_2_ domain can compensate the reduction of DNA binding in high salt condition (Gao et al., 2021). Apart from its weak binding in high salt conditions, Phi29 polymerase possesses potent 3'–5′ exonuclease activity for proofreading polymerization errors, albeit this activity poses a challenge by degrading oligonucleotide-modified substrates and obstructing polymerase processivity (Del Prado et al., 2019; Sun et al., 2023). Strong exonuclease activity and weak salt tolerance have impeded the widespread application of phi29 polymerase in polymerase-nanopore sequencing systems.

Bst DNA polymerase is a thermostable enzyme from *Bacillus stearothermophilus* N3468 and has emerged as an alternative candidate for the same purpose. While exhibiting strong strand displacement and efficient incorporation of modified substrates without 3′–5′ exonuclease activity, this enzyme mandates an elevated DNA amplification temperature of 60°C (Aliotta et al., 1996; Hafner et al., 2001). However, long-read sequencing systems such as Oxford Nanopore or PacBio currently operate at room temperature (Nurk et al., 2022). These sequencing systems typically require several hours of sequencing time, which may pose a challenge to the delicate microenvironment of a nanopore sensor in high temperature conditions. This subsequently directs our attention to identifying new polymerases that meet the characteristic requirements for long read nanopore sequencing.

Psychrophilic polymerase has garnered our interest owing to its isothermal amplification temperature, which is significantly lower than that of thermophilic polymerases (e.g., Bst polymerase) and reminiscent of that observed in mesophilic polymerases, such as phi29 (Xue et al., 2021). Currently, there is no systematic functional mechanism analysis available for psychrophilic polymerases and only two enzymes have been documented. PB polymerase with a D422A mutant site, originating from the marine psychrophilic bacterium *Psychrobacillus* sp. reportedly exhibits enhanced strand-displacement activity and isothermal amplification ability (Piotrowskiet al., 2019). Nevertheless, its amplification is hindered by elevated salt conditions exceeding 150 mM KCl, and the presence of potential exonuclease activity of PB polymerase remains unclear. Another PIPI-WT polymerase from *Psychromonas ingrahamii* reportedly completes the isothermal amplification reaction at temperatures of 30°C–40°C (Xue et al., 2021). However, whether it can meet all the required criteria remains elusive.

In this report, we systematically analyze the functional mechanism of psychrophilic polymerases focusing on their exonuclease activity, salt tolerance, processivity and incorporation using artificial substrates. This fundamental study is instrumental in expediting the development of protein engineering applications in the realm of next-generation nanopore sequencing technologies.

## 2 Materials and methods

### 2.1 Plasmid construction

The sequence of PB polymerase is from a previous report (Piotrowski et al., 2019). Based on this, PB polymerase and PB-mutant variants (PB-422, PB-DXE PB-822, PB-732, and PB-Bst) were synthesized and cloned into the Pet21a plasmid by GenScript (China). Phi29 wild-type and its mutant, ZG_O were also synthesized by GenScript. The His-tag was fused in the C-terminus of these proteins.

Regarding the fusion polymerases, the DNA-binding domain (DBD) from *Pyrococcus abyssi* DNA ligase and the HI domain from helix-hairpin-helix [(HhH)_2_] domain from *Methanopyrus kandleri* topoisomerase V were fused to the N-terminus of PB-Bst polymerase, while the sulfolobus 7-kDa protein (Sso7d) domain from the thermophilic archaea *Sulfolobus solfataricus* was fused to the C-terminus of PB-Bst polymerase by BGI Genomics (Shenzhen, China). PB-E, PB-F, PB-G, and PB-H were provided by Generay Biotechnology Company (Shanghai, China). The His-tags used for purification are found in the C-terminus of all the proteins.

### 2.2 Protein expression

All protein expression plasmids were transformed into chemically competent BL21 *Escherichia coli* cells (ToloBio, Wuxi, China) as per the manufacturer’s instructions. A single colony was cultured in 5 mL of LB medium supplemented with kanamycin (50 μg/mL) overnight at 37°C. This culture was further incubated in 200 mL of liquid LB medium at 37°C on a shaker at 200 rpm until an OD_600_ value of 1 was achieved. Protein expression was induced by adding isopropyl-D-1-thiogalactopyranoside (IPTG) up to a concentration of 0.6 mM (A100487, Sango Biotech, Shanghai, China). The incubation temperature was reduced from 37°C to 20°C, and the protein production was carried out for 24 h. The cells were centrifuged at 8,000 rpm for 10 min, collected, and then stored at −80°C until further use.

All protein purification steps were performed at 4°C. The cells were resuspended in 40 mL of binding buffer (50 mM Tris-HCl [pH 7.5] and 500 mM NaCl) and sonicated (2 s ON and 5 s OFF for 20 min; 01C1503; SCIENTZ, NingBo). After centrifugation at 12,000 rpm for 30 min, the soluble protein was collected. The expressed His-tagged target proteins were purified using a Ni Sepharose 6FF (17531801, Cytiva) gravity column. The target protein was eluted using a binding buffer (50 mM Tris-HCl [pH 7.5] and 500 mM NaCl) containing different concentrations of imidazole, and the target protein was detected via 10% SDS-PAGE. High-purity elution components were subjected to 10 K ultrafiltration (157655 (24), Millipore) to remove imidazole and obtain a high concentration of target proteins.

### 2.3 Structural analysis

Polymerase structures were generated using the AlphaFold2 Colab notebook (Cramer, 2021; van Breugel et al., 2022), and amino acid stability analysis was performed using HotSpot Wizard 3.0, to obtain information about amino acid recurrence rate and potential catalytic activity after mutation (Sumbalova et al., 2018). Using HotSpot Wizard 3.0, their mutation scores were determined to facilitate the choice of amino acids for specific positions.

### 2.4 Exonuclease activity

The exonuclease activity of chimeric PB DNA polymerases was detected using a non-thiolated M13mp18 ssDNA primer. Briefly, 1 µM primer and 200 nM polymerase were mixed in a 1 × phi29 buffer (B0269S, New England Biolabs, Cambridge, UK) and adjusted to a final volume of 20 µL with double-distilled water. The reaction was performed at 30°C for 30 min and analyzed via electrophoresis on a 10% TBE-Urea gel (EC68752BOX, ThermoFisher).

The oligonucleotides were modified with two constitutive phosphorothioates (CGCCAGGGTTTTCCCAGTCACGA^s^C^s^) (Supplementary Figure S1) or two constitutive C3 spacers (CGC​CAG​GGT​TTT​CCC​AGT​CAC​GA^c^C^c^) at the 3′ end as a protecting group. These were exploited as substrates to further determine the obstructive effect of the protecting group on exonuclease activity. The method of testing exonuclease activity was the same as that using non-thiolated M13mp18 ssDNA primers as substrates.

To address how salt could affect exonuclease activity, 100 nM of non-thiolated M13mp18 ssDNA primer or oligo-modified dGTP (Supplementary Figure S1) were mixed with 200 nM of the polymerase proteins under salt-free and 300 mM KCl conditions for 10 and 150 min. The results were tested via electrophoresis on a 10% TBE-Urea gel.

### 2.5 Rolling circle amplification (RCA)

The template–primer complex was prepared by combining 200 nM of M13mp18 single-stranded DNA (N4040S, New England Biolabs, Cambridge, United Kingdom) and 1 μM of the primer. M13mp18 was a 7429-nt sscDNA annealed using a specific primer (5′ -CGC​CAG​GGT​TTT​CCC​AGT​CAC​GAC- 3′) at 95°C for 3 min in a 1 × phi29 Pol reaction buffer (B0269S, New England Biolabs, Cambridge, United Kingdom) followed by incubation at room temperature for 25 min.

To understand how various template and polymerase protein molar ratios could affect salt tolerance, polymerases with different final concentrations and 20 nM of M13mp18 were mixed to a total reaction volume of 20 µL containing 50 μM of dNTPs, 1 × phi29 Pol reaction buffer, and 0.2 μg of BSA (B9000S, New England Biolabs, Cambridge, United Kingdom).

To explore polymerase salt tolerance and processivity, 20 nM of M13mp18 was mixed with 200 nM of polymerase proteins under various salt concentration conditions for different durations. The mixtures were reacted at 30°C for 180 min, and then the reaction was terminated by adding 0.5 M EDTA. The RCA assay was repeated for every polymerase thrice.

In these RCA assays using oligonucleotide–modified substrate, the polymerases and templates were mixed at a molar ratio of 20:1. The reaction mixture included 200 nM of polymerase proteins, 10 nM of the M13mp18 template–primer complex, 1 × phi29 buffer, and 0.2 μg BSA. The oligonucleotide-modified substrate was added at a final concentration of 50 μM, while the other three natural nucleotides were maintained at a final concentration of 5 μM. The total reaction volume was adjusted to 20 μL using double-distilled water. The reaction was carried out at 30°C for 3 h. All the oligonucleotide-modified substrates were provided by AXBIO (Axbio Biotechnology (Shenzhen) Co. Ltd. Shenzhen, China).

The RCA product was analyzed by 0.6% alkaline agarose gel electrophoresis. The GeneRuler High Range DNA Ladder (10.1–48.5 Kb, SM1351; Thermo Fisher Scientific, United States) and 1 Kb Plus DNA Ladder (0.3–10 kb, P21119, TransGen Biotech Co. Ltd., Beijing, China) were used as DNA molecular weight markers. A peristaltic pump (BT100-1 L; LongerPump Co. Ltd., Baoding, China) was used to circulate the running buffer to avoid high temperatures while running the gel. After 2–3 h of electrophoresis, the gel was stained with SYBR Gold, and the concentrations and molecular weights of the RCA product were assessed by AzureSpot.

## 3 Results

### 3.1 Regulatory mechanism analysis of exonuclease activity in psychrophilic polymerase

**FIGURE 1:**
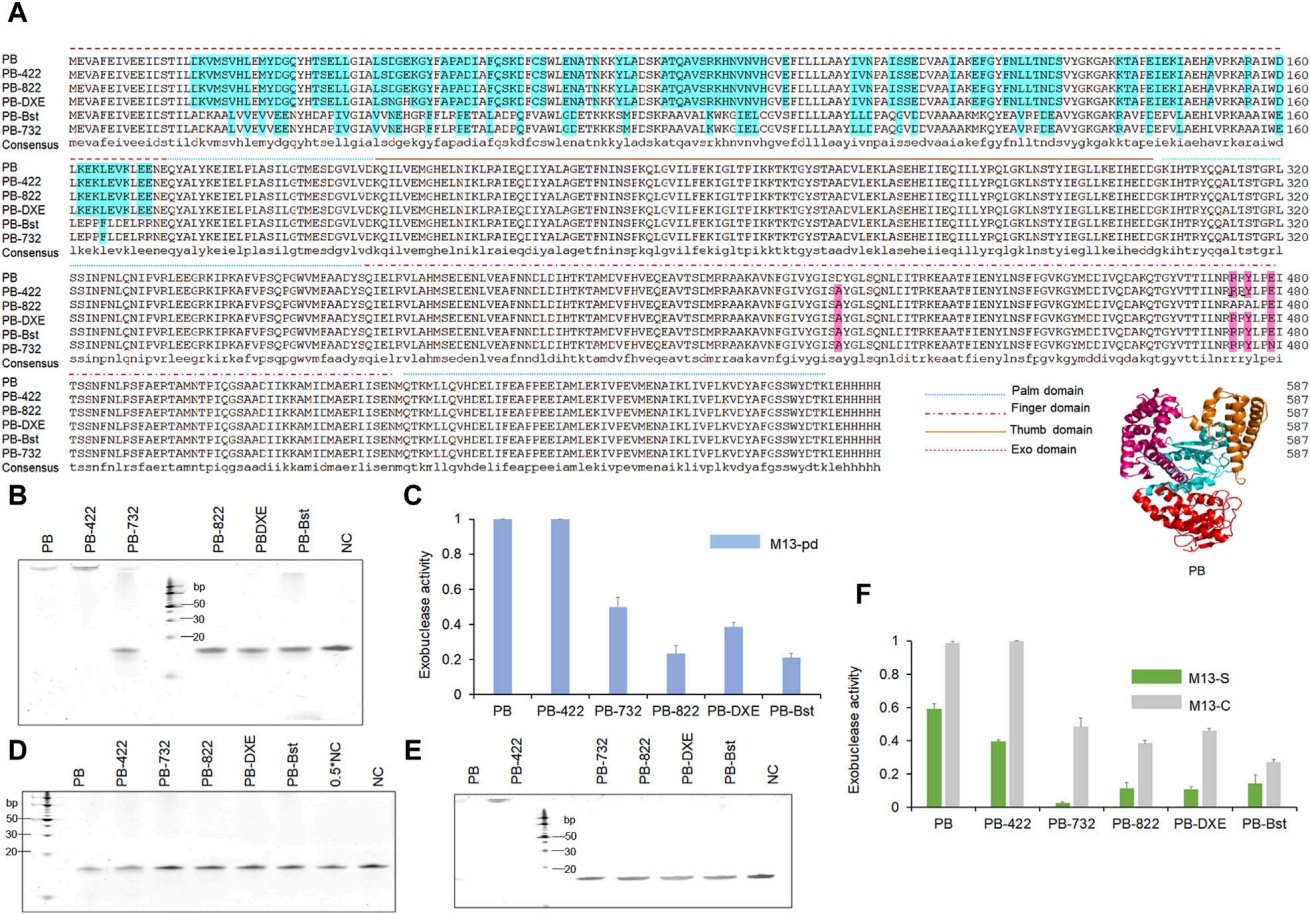
Sequence alignments and 3′–5′exonuclease activity analysis of the PB mutant variants polymerases. **(A)** Polymerase sequence alignments of PB polymerase mutant variants. Different line modes represent the palm, finger, thumb, and exonuclease domains of the polymerases. **(B, C)** Decreasing exonuclease activity of PB polymerase mutant variants. 200 nM polymerase was incubated with 1 μM oligonucleotides (M13pd) for 30 min, followed by TBE gel analysis **(B)** and quantification using ImageJ **(C)**. **(D–F)** The modified phosphorothioate or C3 spacer at the 3′-end reduced oligonucleotide degradation. A total of 200 nM of polymerase and 1 μM of oligonucleotides M13-S **(D)** or M13-C **(E)** was mixed for 30 min, followed by TBE gel analysis and quantification using ImageJ **(F)**. Levels were normalized to the negative control (NC). NC refers to only M13-S or M13-C. Data are presented as mean ± SD.

To test the exonuclease activities of PB psychrophilic polymerase, we incubated 200 nM polymerase with 1 μM oligonucleotide (M13pd) for 30 min. Based on our TBE gel analysis, both PB wild-type polymerase and PB with D422A polymerase (PB-422) exhibited strong exonuclease activity. During polymerase-nanopore sequencing, the strong exonuclease activities of PB and PB-422 polymerase would degrade oligonucleotide-modified substrates, resulting in the reduction of electrochemical signals. Therefore, to explore the regulatory mechanism of exonuclease activity and eliminate this activity, we engineered PB polymerase based on the predicted structure analysis and bibliographic retrieval regarding the exonuclease activity of other polymerases. PB polymerase is a family A polymerase. This family is characterized by three conservative exonuclease domains, including the DXE, NX_2_3_(F/Y)D, and YX_3_D motifs (Park et al., 1997; Czernecki et al., 2023), of which the first could be detected in PB polymerase. We mutated DGE (Asp43, Gly44, and Glu45 in PB polymerase) to NGH (D43N and E45H in Bst polymerase) based on the sequencing alignment of Bst and PB polymerase, as the first mutant variant, PB-DXE. The RRRY motif in the Klenow fragment polymerase reportedly plays an important role in the 3′–5′ exonuclease activity, and R822A/Y824A in the RRRY motif exhibited better polymerase activities and weaker exonuclease activities than the wild-type of Klenow fragment polymerase (Kukreti et al., 2008). Inspired by this, we obtained the second mutant variant (R474A and Y476A), named PB-822. Bst polymerase, as a family A polymerase, does not display any exonuclease activity (Aliotta et al., 1996). The PB polymerase exonuclease domain was replaced with that of Bst polymerase, namely, PB-Bst, as the third mutant variant, to eliminate the exonuclease activity. Taq DNA polymerase also reportedly exhibited strand-displacement activity and faster PCR processing ability by a single-site mutant, namely, D732N (Barnes et al., 2020). We introduced this site into the PB-Bst polymerase (E479N) as the fourth mutant variant, PB-732, to tip the balance between the exonuclease and polymerase activities. Their sequences and functional domains are summarized in Figure 1A.

Through the following exonuclease activity test, we revealed that PB-822, PB-DXE, and PB-Bst were almost unable to degrade the oligonucleotide (Figure 1B). In particular, the exonuclease activity of PB-822 and PB-Bst were significantly reduced to less than 25% (Figure 1C). However, we also observed that E479N reversed the exonuclease activity in PB-732 polymerase (more than 40%) compared with PB-Bst polymerase (<25%). Hence, functional structure domain replacement is an efficient strategy to engineer proteins for obtaining potential functions.

To explore alternative strategies for safeguarding oligonucleotides against degradation, we synthesized two additional oligonucleotides modified with two constitutive phosphorothioates or constitutive C3 spacers at the 3′ end. These modifications have been reported to impede the exonuclease activity of VpV62 polymerase, preventing the degradation of oligonucleotides (Gao et al., 2020). Nevertheless, their efficacy to function in PB polymerase remains uncertain. Through the exonuclease activity test, we observed that although PB-422 or PB wild-type displayed strong exonuclease activity, the two constitutive phosphorothioate modifications could significantly inhibit oligonucleotide degradation, while the two constitutive C3 spacer modifications could not (Figures 1D,E). Furthermore, the two constitutive phosphorothioate modifications demonstrated an additional capability to further diminish oligonucleotide degradation when employed PB-732, PB-822, PB-DXE, and PB-Bst polymerases (Figure 1D). The two constitutive C3 spacer modifications resulted in an effect such as that of natural oligonucleotides for PB-732, PB-822, PB-DXE, and PB-Bst polymerases (Figures 1C,F). Consequently, the two constitutive phosphorothioate modifications (Supplementary Figure S1) were deemed as an optimal method for mitigating exonuclease activity-induced degradation.

### 3.2 Structural analysis of psychrophilic polymerase exonuclease activity

The exonuclease activity of polymerases is a key limitation for their utilization in nanopore sequencing system due to its degradation of oligo-modified substrates. PB-Bst did not show exonuclease activity. However, Phi29 polymerase as a commonly known polymerase is limited in nanopore sequencing system due to its strong exonuclease activity (Supplementary Figure S2A). Therefore, we employed AlphaFold2 and PyMOL for protein structure prediction and analysis of phi29 and PB-Bst to explore their exonuclease activity regulatory mechanisms (Cramer, 2021). Through generating and visualizing the electrostatic potential distribution, a crucial insight emerged: a basic structural protrusion, resembling two fingers, situated at the periphery of the exonuclease active pocket proved to be indispensable for exonuclease activity in phi29 polymerase (indicated by the red arrow, Supplementary Figure S2B). In the context of this study, the specific structural protrusion is addressed to as “finger protrusion”. We postulated that the finger protrusion with a basic property, functions in guiding the single-stranded DNA (ssDNA) into the exonuclease active center, and the removal of this would result in the elimination of exonuclease activity.

To further validate our hypothesis, we modified phi29 polymerase, specifically aiming to eliminate its basic finger protrusion at the periphery of the exonuclease active pocket. Asp12, Glu14, Tyr59, His61, Asn62, Asp66, Phe69, Lys143, Tyr148, Tyr165, and Asp169 are reported to directly interact with ssDNA, regulating the exonuclease activity (Esteban et al., 1994; Eisenbrandt et al., 2002; Perez-Arnaiz et al., 2009). Notably, D12A and D66A have been demonstrated to completely abolish the exonuclease activity of phi29 polymerase (Del Prado et al., 2019) and we showed the consistent result (Supplementary Figure S2A). However, the impact of the specific finger protrusion on phi29 polymerase’s exonuclease activity has not been elucidated. We incorporated mutagenic alterations V141K and K143G into the phi29 polymerase as ZG_O (Supplementary Figure S2B). The K143G mutation removed this specific structural protrusion while V141K preserved the basic property of this position. Both mutations eliminated the physical obstruction of the finger protrusion without altering the charge. In agreement with our hypothesis, the exonuclease assay revealed that V141K and K143G significantly attenuated the degradation ability of phi29 polymerase compared with phi29-wild-type (Supplementary Figure S2C), confirming the essential role of the basic finger protrusion in regulating phi29 polymerase exonuclease activities. However, more experiments were required to clarify the role of the charge in the finger protrusion for phi29 polymerases and the function of basic finger protrusion in other polymerases.

**FIGURE 2:**
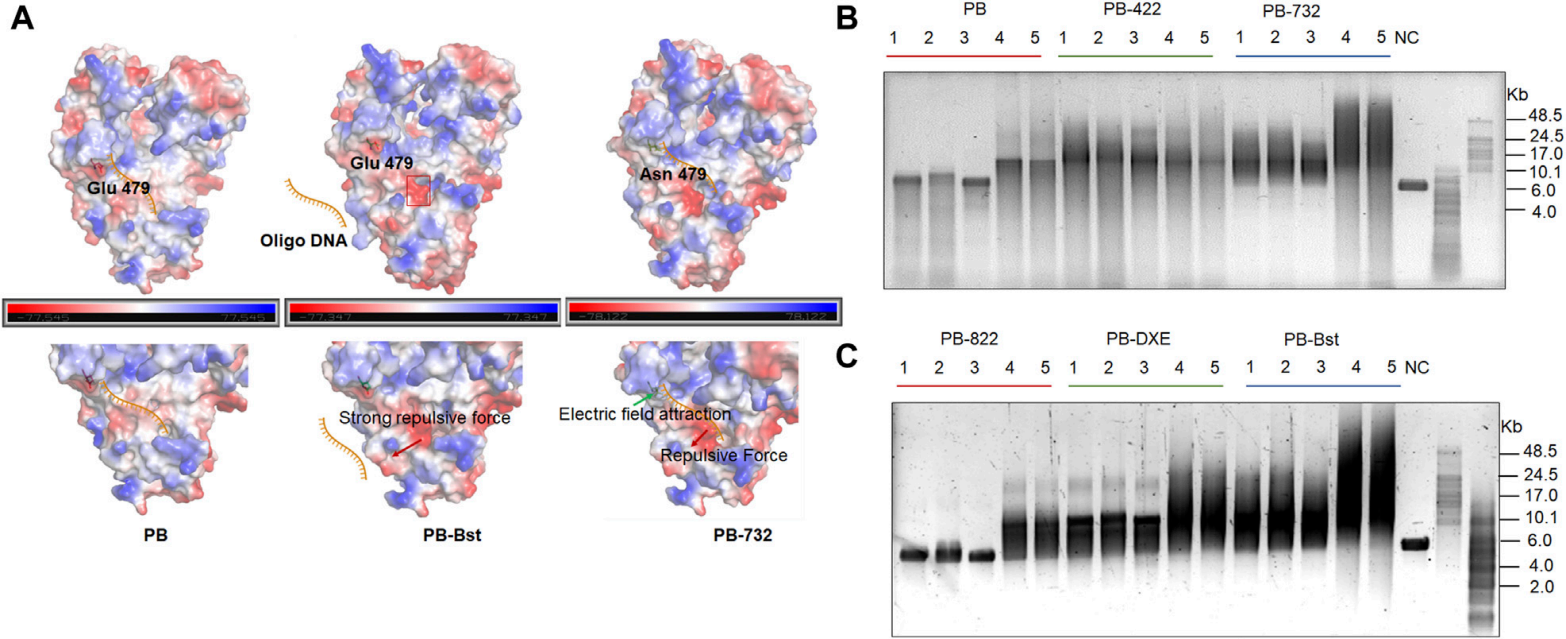
Structural analysis of exonuclease activity, and salt tolerance analysis of polymerase mutant variants. **(A)** Charged surface analysis of PB polymerases using PyMOL. Green and red arrows indicate the attractive and repulsive forces. Red frame refers to a pronounced acidic edge in close proximity to the exonuclease pocket. **(B, C)** RCA assays of PB polymerase mutant variants using M13mp18 DNA as a template. Lanes 1–3 refer to different template to polymerase molar ratios under high salt conditions as follows: lane 1 (10 nM vs. 400 nM); lane 2 (10 nM vs. 800 nM); lane 3 (10 nM vs. 200 nM). Lanes 4 and 5 refer to different template-to-polymerase molar ratios without extra salt: lane 4 (10 nM vs. 400 nM); lane 5 (10 nM vs. 800 nM).

Furthermore, we identified another distinctive protein structure, characterized by a pronounced acidic edge in close proximity to the exonuclease pocket in PB-Bst (Figure 2A, red frame). Here, we hypothesize that this prominent acidic edge, with its strong positive charge, generates a repulsive force that complicates the entry of ssDNA into the exonuclease active center, ultimately leading to the elimination of exonuclease activity (indicated by the red arrow, Figure 2A). Bst polymerase and PB-Bst fusion polymerase, known to be devoid of exonuclease activity (Figure 1C and Supplementary Figure S2A), was structurally scrutinized in comparison with PB. PB-Bst polymerases, which retains the exonuclease domain of Bst polymerase, maintained a robust acidic edge, a feature absent in PB. This observation aligns with our hypothesis that a more pronounced acidic edge in the vicinity of the exonuclease pocket is vital for exonuclease activity. Interestingly, we also observed that E479N partly restored the exonuclease activity in PB-Bst polymerase. This may be due to E479N increasing positive charge in PB-732, resulting in a stronger binding affinity of oligonucleotides to perform exonuclease activity (Figure 2A, green arrows).

Consequently, two distinct methodologies were developed to validate our theory of obstructing ssDNA from entering the exonuclease activity center, serving as a means of eliminating the exonuclease activity. The first approach involves the removal of the basic structural protrusion, and the second method aims to enhance a potent acidic edge for effective strand displacement.

### 3.3 Improving the salt tolerance of psychrophilic polymerase

To begin with, different reaction buffers were tested to determine the optimal psychrophilic polymerase buffer. PB-wild-type and PB-422 amplified longer products in buffer 1 than in the previous PB buffer which was recommended by Piotrowski (Piotrowski et al., 2019) (Supplementary Figure S3). Moreover, both enzymes could amplify in buffer 1 with 300 mM KCl, but not in the recommended PB buffer with 300 mM KCl (Supplementary Figure S3). As a result, buffer 1 (50 mM Tris-HCl, 10 mM MgCl_2_, 10 mM (NH4)_2_SO_4_, 4 mM DTT, pH 7.5 @ 25°C) was more effective than the recommended PB buffer (50 mM BIS-Tris propane, pH 8.5 @ 25°C, 5 mM MgCl2, 1 mM DTT, and 2% glycerin). PB-422 displayed improved processivity under different salt concentration conditions compared with PB-wild type. This result was consistent with a previous report in which Asp422 was substituted by Ala422, resulting in increased strand-displacement activity. Buffer 1 was then chosen for the follow-up extension experiments of all psychrophilic polymerases.

To validate the salt tolerance of different variants, RCA was performed under different salt concentration conditions with varied template and polymerase molar ratios. All PB polymerase mutant variants displayed an increase in processivity, and salt tolerance compared with PB-wild-type except for PB-822 (Figures 2B,C). The RCA bands of PB-422 were more dispersive, while both PB-732 and PB-Bst exhibited higher extension rates and stronger processivity activities than all others under either low or high salt conditions (Figures 2B,C). As PB-732 did not yield a longer product than PB-Bst, E479N was not a favorable mutant site to improve PB-Bst polymerase processivity. The PB-Bst product size ranged 8–24 Kb under high salt condition. PB-822 can amplify under salt-free conditions but not at 300 mM KCl conditions. Some very weak bands were detected in the 300 mM KCl sample only when using a template-to-PB-822 M ratio exceeding 80 times (Figure 2C). The RCA product of PB-DXE was centralized under the 300 mM KCl conditions in the range of 7–15 Kb and observed a distinctive longer band of approximately 24 Kb. Higher template-to-polymerase molar ratios (1:40 and 1:80) did not yield significantly improved processivity and stronger salt tolerance compared with the lower ratio (1:20) for the series of PB mutant variants (Figures 2B,C). In conclusion, substituting the exonuclease domains in PB polymerase not only eliminated its exonuclease activity, but also enhanced its salt tolerance.

We also compared phi29 polymerase, exo^−^-phi29 polymerases (mutated sites D12A and D66A) and Bst polymerases with PB-Bst polymerases (Del Prado et al., 2019). At room temperature, Bst and exo^−^-phi29 were unable to amplify under 300 mM KCl condition, whereas PB-Bst and wildtype phi29 exhibited amplification capabilities. Notably, the loss of exonuclease activity in phi29 polymerase resulted in a significant reduction in its processivity (Supplementary Figure S4).

All of them facilitates PB-Bst as a suitable substitution for phi29 polymerase in nanopore sequencing.

### 3.4 Improving the processivity of psychrophilic polymerase

Improving processivity will assist nanopore sequencing with longer read lengths, further facilitating transcript isoform identification and genetic variant detection. Hence, to achieve improved processivity, we proceeded to engineer these polymerases as follows.

**FIGURE 3:**
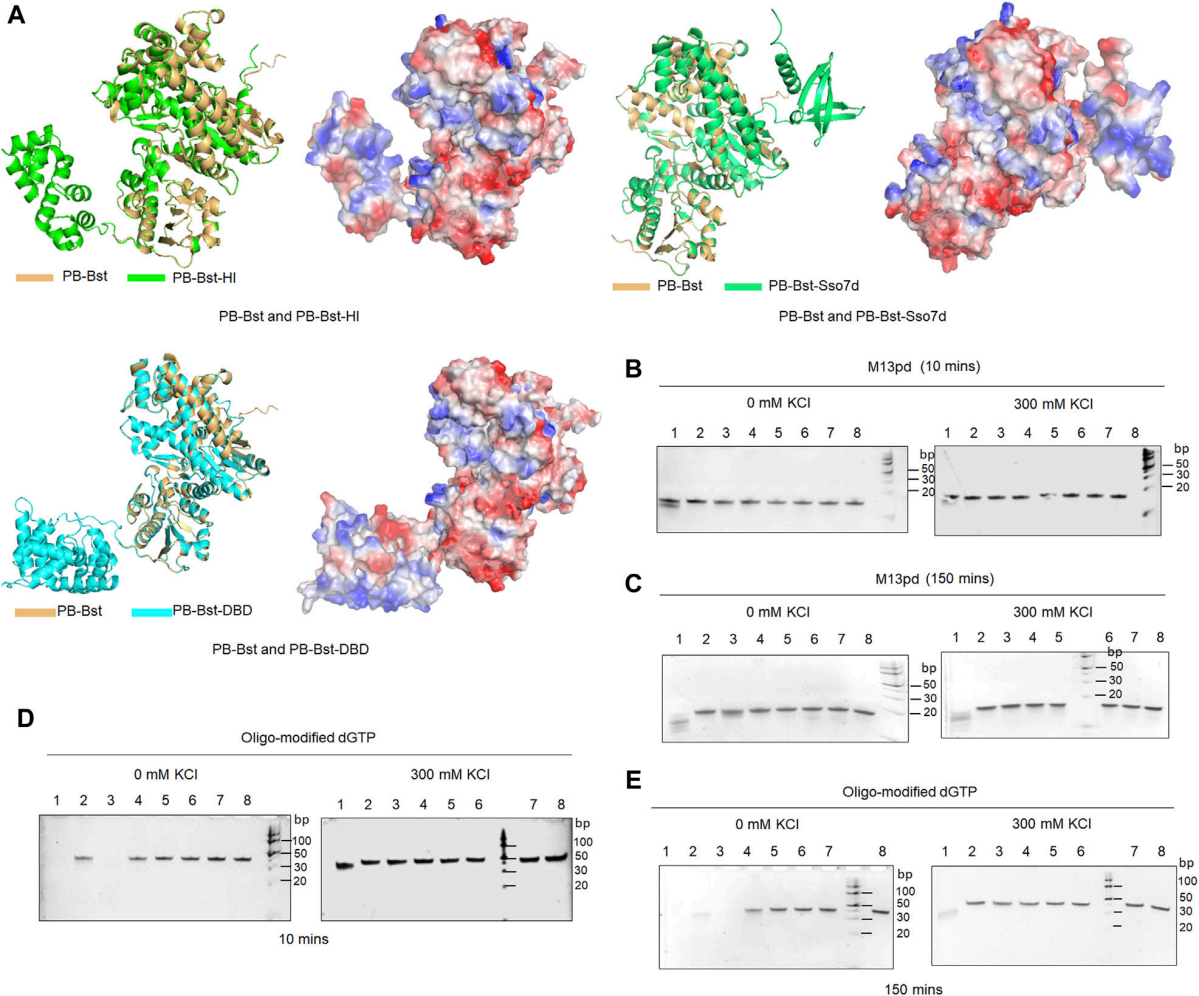
3′–5′exonuclease activity analysis of PB fusion polymerases. **(A)** Structures of PB-Bst, PB-Sso7d, PB-DBD, and PB-HI polymerases determined using AlphaFold2, along with their charged surfaces visualized using PyMOL; **(B, C)** Each of the polymerase (200 nM) was mixed with 1 µM of oligonucleotides (M13pd) separately for 10 and 150 min under different salt concentration conditions. **(D, E)** Oligo-modified substrate was further used to test 3′–5′ exonuclease activities of the PB polymerase mutant variants and fusion of PB polymerase under different time and salt concentration conditions. Lanes 1–7 refer to PB-422, PB-DXE, PB-822, PB-Bst, PB-HI, PB-Sso7d and PB-DBD, respectively. Lane 8 refers to M13pd; the reactions were analyzed using a 10% TBE-urea gel.

For the PB polymerase mutant variants, the largest fragment of the RCA product ranged 8–24 Kb in 300 mM KCl. To improve their extension ability, three specific functional domains were considered to be fused with PB-Bst polymerase. Sulfolobus 7-kDa protein, Sso7d was the first considered functional domain from the thermophilic archaea *S. solfataricu*; it has been reported to improve Pfu-polymerase processivity through stabilizing the DNA and polymerase complex (Eom et al., 2004). DNA-binding domain (DBD) from *P. abyssi* DNA ligase was the second considered protein domain and is an independently folded protein domain to enhance DNA binding affinity. DBD could help Taq polymerase eliminate the inhibitory effect of heparin (Oscorbin et al., 2015). Helix-hairpin-Helix [(HhH)_2_] domain from *M. kandleri* topoisomerase V has been successfully fused to Taq DNA polymerase and Pfu DNA polymerase to increase their salt tolerance and processivity (Pavlov et al., 2002). Different HhH motifs might affect the extension rates differently. Coupling the H and I motifs from the [(HhH)_2_] domain with phi29 polymerase significantly enhanced its DNA binding affinity (de Vega et al., 2010). The Sso7d, DBD, and HI domains were fused to PB-Bst to analyze how these functional domains could affect psychrophilic polymerase processivity, followed by predicting their structures using AlphaFold2 (Figure 3A).

Initially, an exonuclease assay was performed to examine the exonuclease activity of these fusion polymerases. The DBD and HI domains were located at the N-terminus while Sso7d at the C-terminus. PB-Sso7d, PB-DBD, and PB-HI did not exhibit strong exonuclease activities under salt-free and high salt conditions for both 10 and 150 min (Figures 3B,C). The high salt conditions not only inhibited the processivity of PB-422, PB-DXE, and PB-Bst polymerases (Figures 2B,C) but also limited their exonuclease activities (Figure 3). In particular, PB-DXE, PB-Bst, PB-Sso7d, PB-DBD, and PB-HI resulted in very low exonuclease activity in 300 mM KCl for 150 min (Figure 3C). Polymer-tagged nucleotides are commonly used as sequencing substrates in long-read nanopore sequencing systems (Fuller et al., 2016), although these oligonucleotide modifications in artificial substrates can be degraded by the exonuclease activity of the polymerases. To further determine the degradation effect of these PB-fusion polymerases on polymer-tagged nucleotides, oligo-modified dGTP was synthesized (Supplementary Figure S1). PB-422 quickly degraded oligo-modified dGTP under salt-free condition for 10 min, but high salt conditions significantly inhibited this degradation process (Figure 3D). Although PB-822 did not exhibit significant exonuclease activity for natural oligonucleotides (Figure 3B), it notably degraded oligo-modified dGTP under salt-free conditions (Figure 3D). However, in the presence of 300 mM KCl, the exonuclease activity of PB-822 was considerably inhibited (Figure 3E). On the other hand, PB-Bst, PB-Sso7d, PB-DBD, and PB-HI did not degrade oligo-modified dGTP, either in 0 mM KCl or 300 mM KCl, during both 10- or 150-min intervals (Figures 3D,E).

**FIGURE 4:**
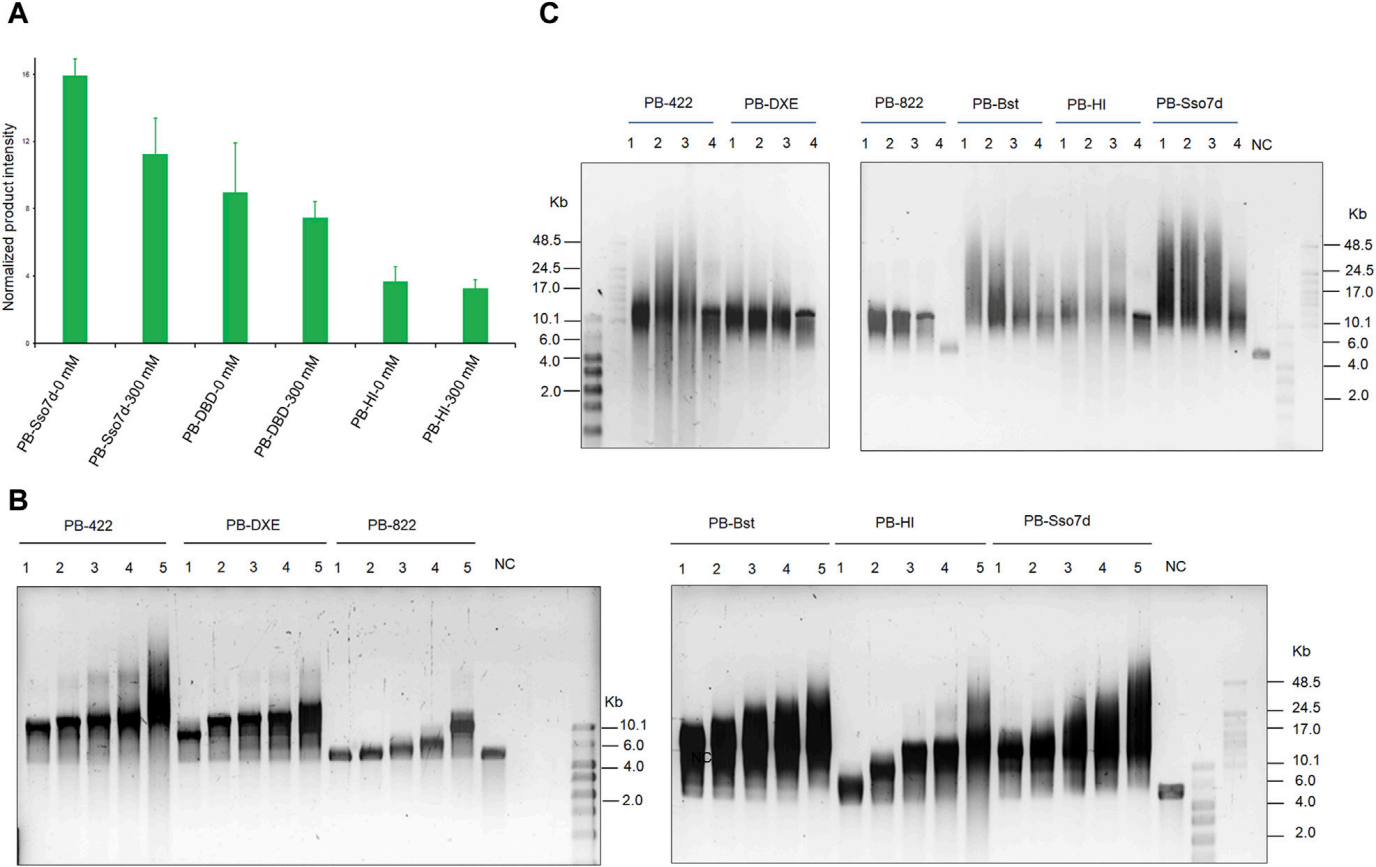
Improving processivity activity of psychrophilic polymerase. **(A)** RCA product quantifications of fusion PB DNA polymerase performed using ImageJ. Replication of 20 nM of primed M13mp18 DNA was performed using 200 nM of the fusion PB DNA polymerases. Levels were normalized to the negative control (RCA reagent without polymerases). Data are presented as mean ± SD. **(B)** RCA analysis of chimeric and fusion PB DNA polymerases for various times. Lanes 1–5 refer to 30, 60, 120, 180, and 360 min, respectively; NC refers to template only. **(C)** Strand displacement coupled M13mp18 DNA replication by chimeric PB DNA polymerases and fusion PB DNA polymerases under different salt concentration conditions. Lanes 1 – 4 refer to salt-free conditions, 150 mM KCl, 200 mM KCl, and 300 mM KCl, respectively. NC indicates template without polymerase. The reactions were analyzed using a 0.6% alkaline agarose gel.

Next, an RCA assay was performed to investigate how the fusion domain affects polymerase processivity. Sso7d significantly improves PB polymerase processivity when compared to the DBD and HI domains under salt-free and high salt conditions (Figure 4A). By considering the product intensity results, the processivity of PB polymerase mutant variants was tested under different time and salt concentration conditions. The RCA product length time-gradient-dependently extended under 300 mM KCl conditions (Figure 4B). In the first 30 min, PB-Bst and PB-Sso7d displayed a faster amplification rate (11 bp/s) than the other polymerases. Their speed decreased in a gradient-dependent manner. However, the product of their RCA amplification continued to elongate over time. The size of the PB-422 RCA product was also found to be more dispersive (Figure 4C), consistent with the previously described result (Figure 2B). The processivities of PB-Bst and PB-Sso7d decreased with reducing salt concentration (Figure 4C). Among the psychrophilic polymerases, PB-Sso7d yielded the highest polymerase activity compared with PB-HI, PB-Bst, PB-822, PB-DXE, and PB-422 in 300 mM KCl. Although PB-Bst and PB-Sso7d yielded similar-sized RCA products, the intensity of the RCA product was higher than that of PB-Bst.

We also tested the suitable temperatures for these polymerases. Although these polymerases could replicate at 10°C, their products were particularly weak (Supplementary Figure S5). Until 30°C, these polymerases amplified significantly. The RCA amplification of PB-Bst and PB-Sso7d was enhanced with increasing temperature. In particular, at 40°C, PB-Bst and PB-Sso7d, with exonuclease domain substitution from Bst polymerase, showed stronger processivity than others. 50°C–65°C is the optimal reaction temperature of Bst polymerases. Therefore, we postulated that the structural substitution from the thermophilic Bst polymerase improved PB polymerase thermostability (Supplementary Figures S5, S6).

The DBD and HI domains did not enhance the processivity and salt tolerance for the PB polymerase mutant variants, as indicated by previous studies (Pavlov et al., 2002; Boyarskikh et al., 2015), implying that the HI domain, being thermophiles, is advantageous in mesophilic and thermophilic polymerases, such as phi29 and Taq polymerases. Although the Sso7d domain also derived from thermophilic archaea, it was available for psychrophilic polymerase to improve its processivity and salt tolerance. Similar functional domains from psychrophilic organisms should be explored in the future to further improve psychrophilic polymerase processivity.

### 3.5 Improving the polymer-tagged nucleotide incorporation of psychrophilic polymerase

Polymer-tagged nucleotides are reportedly available substrates for the single-molecule sequencing system (Stranges et al., 2016). As oligonucleotides, polymer-tagged nucleotides could be degraded by the exonuclease activity of polymerases, thereby necessitating polymerases to eliminate their exonuclease activity. PB-Bst, PB-Sso7d, PB-HI, and PB-DBD did not display any significant exonuclease activity compared with PB-422 and PB-wild-type (Figure 3). Whether these engineered polymerases could effectively amplify using polymer-tagged nucleotides remains to be explored.

**FIGURE 5:**
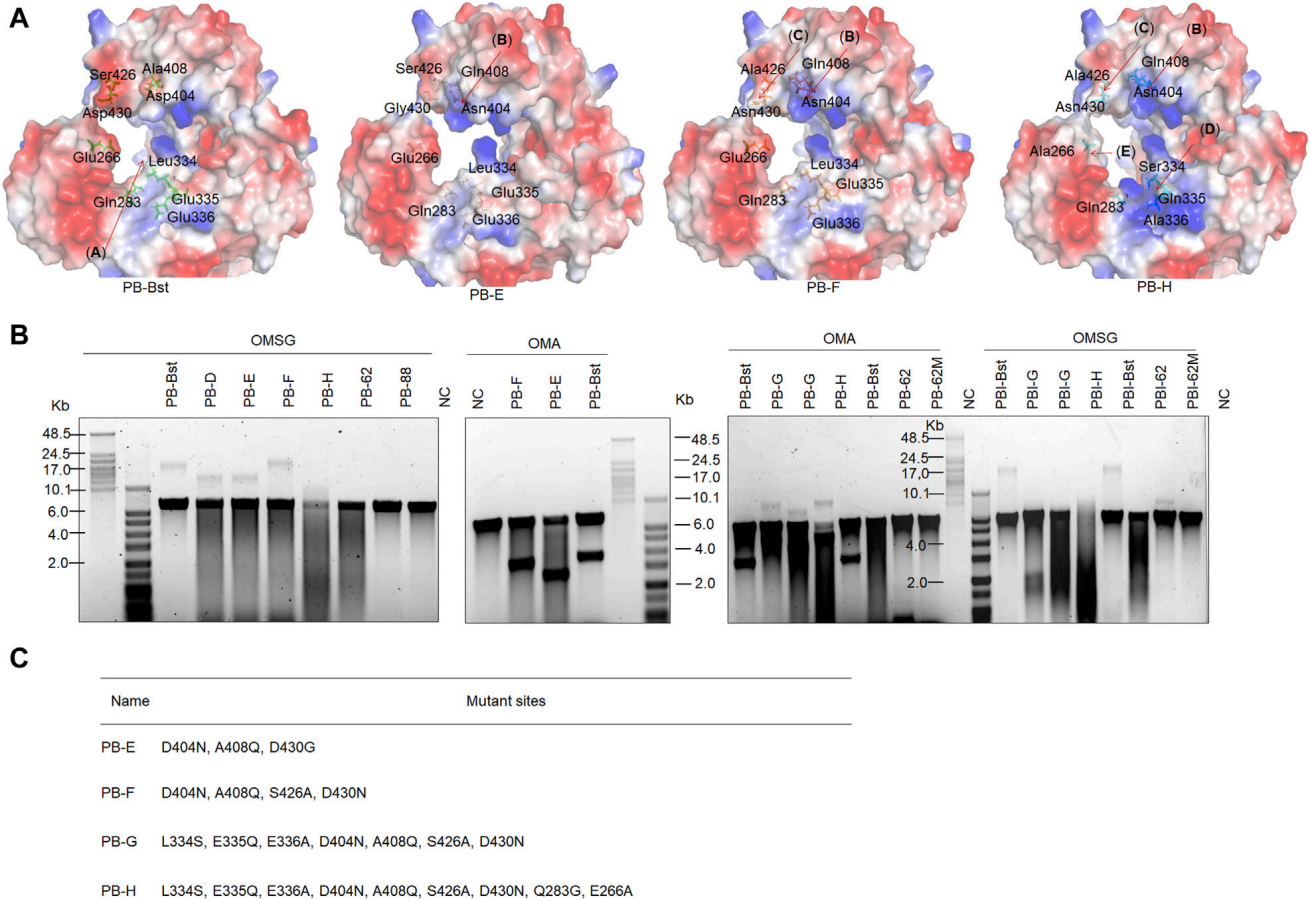
Improving artificial nucleotide incorporation of chimeric PB DNA polymerases. **(A)** Surface charge and mutant site analysis of PB-Bst, PB-E, PB-F, and PB-H; **(B)** RCA analysis of chimeric PB DNA polymerases using an artificial nucleotide as a substitution. Replication of 20 nM primed M13mp18 DNA was performed using 200 nM of chimeric PB DNA polymerases and 50 μM of oligonucleotide-modified substrate at 30°C for 3 h. The RCA products were analyzed using a 0.6% alkaline agarose gel. **(C)** All mutant sites in PB-E, PB-F, PB-G, and PB-H are shown.

Figure 1A shows the functional domains of family A psychrophilic polymerases. The palm domain functions as a polymerase active center. The thumb domain stabilizes the template-primer complex. The finger domain participates in processivity and strand displacement, similar to the TRP2 domain in phi29 polymerase. For polymer-tagged nucleotide incorporation, we proposed that domain (**A**) was the main entrance of the artificial substrate (Figure 5A), consisting of both palm and thumb domains. To explore the regulatory mechanism of the incorporation ability of psychrophilic polymerases using polymer-tagged nucleotides, we engineered PB-Bst polymerase and analyzed their processivity for different polymer-tagged nucleotides. First, we employed two kinds of polymer-tagged nucleotides (oligo-modified short dGTP, OMSG, MW < 10000; oligo-modified dATP, OMA, MW > 10000) to determine the incorporation ability of PB-Bst. Supplementary Figure S1 shows the structures of OMSG and OMA. More than 20 Kb products by PB-Bst with OMSG was amplified in 300 mM KCl, but it was not possible when using OMA under high salt conditions (Figure 5B). The size of the domain entrances might be a key limiting factor for polymer-tagged nucleotide incorporation. Hence, we tried to modify the surface charge and the size of the substrate entrance (domain (**A**)). For Bst polymerase, Gln704 interacted with Asn700 to create strong surface alkalinity (Supplementary Figure S7). This alkalinity was postulated to assist polymer-tagged nucleotide incorporation. We correspondingly mutated Ala408 and Asp404 to Gln and Asn to increase the alkalinity charge in PB polymerase, similar to Bst polymerase (Figure 5A, domain (**B**)). Similarly, Ser426 and Asp430 (Figure 5A, domain (**C**)) were mutated to Ala and Asn to reduce the surface acidity of PB-Bst. Based on the four mutant sites, we designed two mutant variants as PB-E and PB-F. To create a stronger alkaline surface in the domain (**A**) entrance, Leu334, Glu335, and Glu336 in PB polymerase were mutated to Ser, Gln, and Ala, respectively, in PB polymerase (Figure 5A, domain (**D**)) as PB-G. Besides increasing surface alkalinity of the entrance of domain (**A**), enlarging the entrance was another approach to assist in the polymerase incorporation using OMA. Based on PB-G, Gln283 and Glu266 were mutated to Gly and Ala, respectively, to reduce the acidity and physical hindrance (Figure 5, domain (**E**)) as PB-H. All mutant sites are presented in Figure 5C.

As shown in Figure 5B, PB-F exhibited stronger extension than PB-Bst using OMSG under high salt conditions, while PB-E could not yield a longer product than PB-F. This indicated that domain (**C**) (S426 and D430 in PB-F) was vital for the incorporation using polymer-tagged nucleotides such as OMSG, compared with domain (**B**) (A408 and D404 in PB-E). PB-H and PB-G in Figure 5B showed the worst incorporation using OMSG in 300 mM KCl. Based on these results, domain (**D**) (L334S and E335A in PB-G) and domain (**E**) (Q283G and E266A in PB-H) may have inhibited the incorporation of OMSG. However, when OMA was used as a substrate, PB-H showed the best processivity compared with other polymerases under high salt conditions. Domains (**D**) (L334S and E335A) and (**E**) (Q283G and E266A) were important mutant sites for OMA incorporation. PB-F had a similar ability to PB-Bst for OMA incorporation and was better than PB-E. Hence in a polymerase-nanopore sequencing system, four types of polymerase-tagged nucleotides are used, and the combinations of mutant sites are crucial for achieving longer read lengths in nanopore sequencing.

## 4 Discussion

The elegant nanopore sequencing system necessitates polymerases endowed with robust salt tolerance and heightened processivity, especially in the utilization of polymer-tagged nucleotides. In this comprehensive exploration, our focus has shifted to exploring psychrophilic polymerases as viable alternatives to the conventionally employed phi29 and Bst polymerases. We delve into the intricate molecular mechanisms governing exonuclease activity, salt tolerance, and artificial base incorporation ability in psychrophilic polymerases.

**FIGURE 6:**
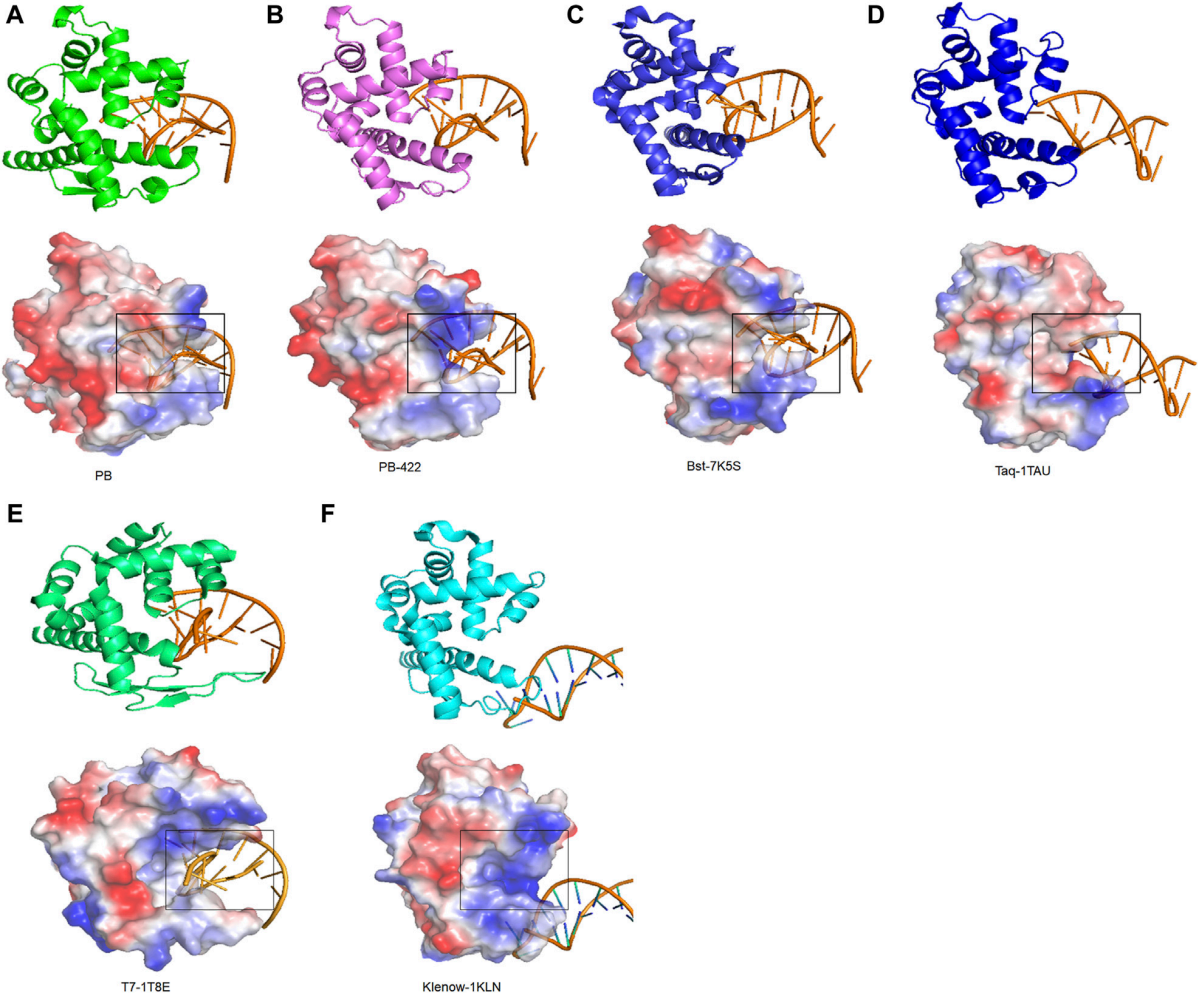
Strand displacement domain analysis of Family A polymerases. Strand displacement domain structures of **(A)** PB, **(B)** PB-422, **(C)** Bst, **(D)** Taq, **(E)** T7 and **(F)** Klenow polymerases determined using AlphaFold2, along with their charged surfaces visualized using PyMOL.

To identify the regulatory mechanisms of strand displacement, the structures of other well-known polymerases from family A, including Taq polymerase (PDB:1TAU), T7 polymerase (PDB:1T8E), Klenow polymerase (PDB:1KLN), and Bst polymerase (PDB:7K5S) were obtained from the PDB database (Beese et al., 1993; Wang et al., 2004; Brieba et al., 2004; Chim et al., 2021). All of them exhibited similar functional domains, consisting of palm, finger, thumb, and exonuclease domains. Based on visualizing electrostatic potential distribution analysis, the same specific semi-circular structure was identified for all, termed as the “ssDNA encapsulation entrance”. Our working hypothesis posits that the presence of an appropriately sized entrance pocket endowed with a robust basic electric charge is indispensable for facilitating effective strand displacement (Figures 6A–F). For example, the ssDNA encapsulation entrance of Taq polymerase was acidic, resulting in the elimination of strand displacement (Figure 6D). Moreover, T7 and Klenow polymerases were also found without strand displacement due to a wide entrance pocket in ssDNA encapsulation entrance (Figures 6E,F). For PB polymerase, the ssDNA encapsulation entrance exhibited a weak alkalinity entrance pocket, resulting in weak strand displacement. D422A in PB-422 polymerases rendered its ssDNA encapsulation entrance more alkaline and less closure to improve the strand displacement of PB polymerase (Figure 2B; Figures 6A,B). In essence, the entrance pocket and electric charge of its ssDNA encapsulation entrance emerges as a vital compartmental structure in governing polymerase strand displacement capabilities.

To pinpoint key regulatory sites for exonuclease activity, salt tolerance, and processivity in psychrophilic polymerases, a series of PB polymerase mutant variants and fusion polymerases were meticulously constructed. D43N and E45H in PB-DXE and R474A and R476A in PB-822, have been validated to attenuate the exonuclease activity of PB polymerase (Figure 1). Interestingly, the presence of the R474A and Y476A mutation in PB-822 had an adverse impact on the processivity of PB polymerase (Figures 2B,C). Through electrostatic potential distribution, R474A and R476A in PB-822 polymerase revealed that an increased acidity in the finger domain resulted in a stronger repulsion force against single-stranded DNA (Supplementary Figure S8). In addition to investigating key amino acid sites for mutation, it is imperative to acknowledge that Bst polymerase, a member of the family A polymerase, has limited exonuclease activity (Aliotta et al., 1996). In this study, we opted for a strategic approach by replacing the exonuclease domain of PB polymerase with that from Bst polymerase, resulting in PB-Bst. PB-Bst exhibited enhanced extension ability and salt tolerance with reduced exonuclease activity (Figure 1; Figure 2; Figure 3). These results emphasize the significance of exonuclease domain substitution as a strategy to engineer proteins for obtaining potential functions.

Processivity, as a crucial polymerase property, determines the read-length of a polymerase-nanopore sequencing system. Fusing functional domains to mesophilic and thermophilic polymerases is an effective strategy to improve their processivity. However, whether this approach is beneficial for psychrophilic polymerase remains unknown. Sso7d, DBD, and HI domains were inserted at the N-, N-, and C-terminus of PB-Bst respectively. Through RCA assays, the HI and DBD domains did not improve the processivity of PB polymerase. We hypothesized that the HI domain from thermophiles is favorable toward mesophilic and thermophilic polymerases such as phi29 and Taq polymerases (Pavlov et al., 2002). However, Sso7d was an available domain for psychrophilic polymerase to enhance its processivity and salt tolerance. Future studies should explore similar functional domains from psychrophilic species to further improve psychrophilic polymerase processivity.

Polymerases were required to employ four different polymer-tagged nucleotides for incorporation in polymerase-nanopore sequencing. The variation in molecular weight among polymer-tagged nucleotides contributed to generating different electrochemical sequencing signals, allowing for the differentiation of the four bases during the sequencing protocol. As presented in Figure 5, PB-Bst yielded >18 Kb DNA products using OMSG; however, it is difficult to take advantage of OMA. We predicted six structural domains, potentially affecting the processivity of polymer-tagged nucleotides. Domain (**C**) (S426 and D430) was important for polymer-tagged nucleotides such as OMSG, where domain (**E**) (Q283 and E266) was necessary for those such as OMA. By combining PB mutant variants with different mutant sites, we can achieve amplification of DNA products exceeding 20 Kb using four different polymer-tagged nucleotides.

In this pioneering investigation, we present the inaugural and comprehensive analysis of regulatory mechanisms governing exonuclease activity, strand displacement, and artificial base incorporation capacity of psychrophilic polymerases. We have successfully identified the key regulatory sites responsible for exonuclease activity (Asp43 and Glu45), and processivity using artificial nucleotides (Gln283, Glu266, Leu334, Glu335, Ser426, and Asp430). A particularly noteworthy finding is the effectiveness of functional domain substitution as a powerful strategy for modifying the functional properties of polymerases, exemplified by the successful elimination of exonuclease activity. Additionally, we also ventured in the fusion of functional domains from thermophilic bacteria to psychrophilic polymerases to enhance their processivity. Notably, the PB-Bst-Sso7d fusion polymerase exhibited robust salt tolerance, improved processivity, and a remarkable absence of exonuclease activity.

This study not only provides valuable insights into the diverse functional properties of polymerases but also introduces novel strategies for their enhancement, including structural domain substitutions, the fusion of additional functional domains, and targeted engineering of key sites within the polymerases. These advancements hold great promise in the field of polymerase-nanopore long-read sequencing technologies, unveiling innovative avenues for further exploration and application.

## Data Availability

The original contributions presented in this study are included in the article/Supplementary Material. Representative AlphaFold2 models are available at https://www.modelarchive.org/. The links were shown in the following list. PB, https://modelarchive.org/doi/10.5452/ma-xsolg; access code: Q6zmNXZ65t. PB-422, https://modelarchive.org/doi/10.5452/ma-f4uwd; access code: iFyMoDSl8o. PB-DXE, https://modelarchive.org/doi/10.5452/ma-0c2l4; access code: bzdY1Oj1Dv. PB-732, https://modelarchive.org/doi/10.5452/ma-ieip7; access code: 8a9mypsqwA. PB-Bst, https://modelarchive.org/doi/10.5452/ma-qjchx; access code: g12HiVM85y. PB-822, https://modelarchive.org/doi/10.5452/ma-t9w9p; access code: F5jQvI4v0z. PB-HI, https://modelarchive.org/doi/10.5452/ma-1bfgy; access code: wqw5Li3r1f. PB-Sso7d, https://modelarchive.org/doi/10.5452/ma-wgecq; access code: pRfomEYZlB. PB-DBD, https://modelarchive.org/doi/10.5452/ma-yig7h; access code: fJochQxKSM. Further inquiries can be directed to the corresponding authors or first author.
