# Supplementary figures and images for: Engineering psychrophilic polymerase for nanopore long-read sequencing
Source: Front Bioeng Biotechnol. 2024 Jul 1;12:1406722. doi: 10.3389/fbioe.2024.1406722 (PMC11246872; doi:10.3389/fbioe.2024.1406722)

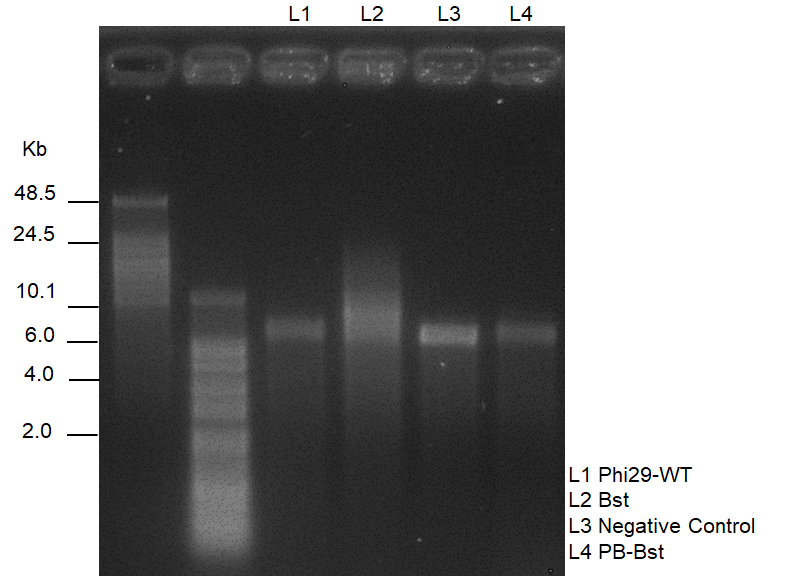

Supplement: Supplementary file 1 [file Image6.TIF]

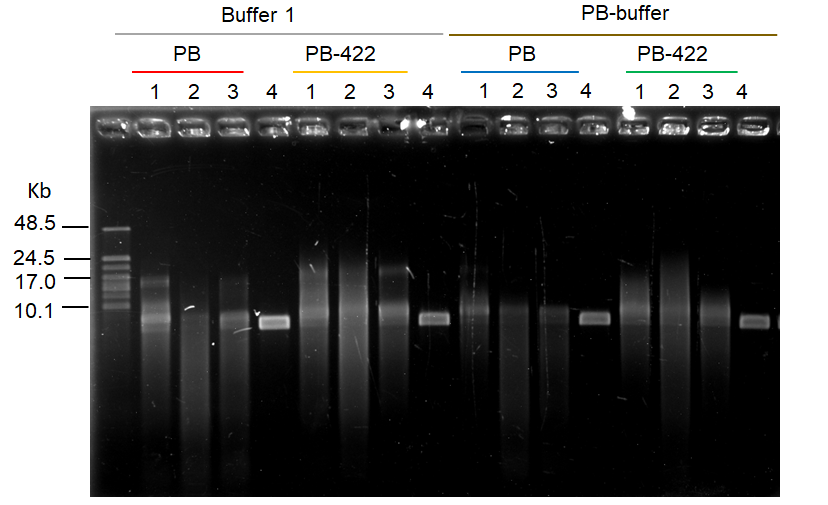

Supplement: Supplementary file 2 [file Image3.TIF]

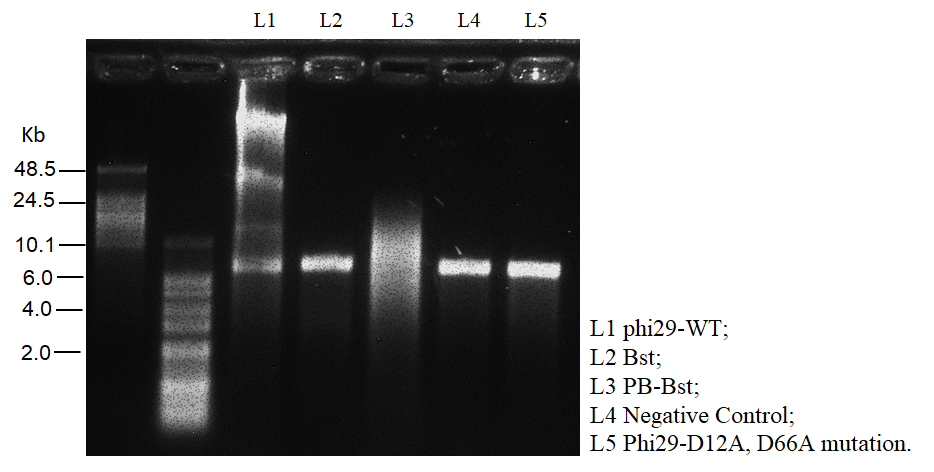

Supplement: Supplementary file 3 [file Image4.TIF]

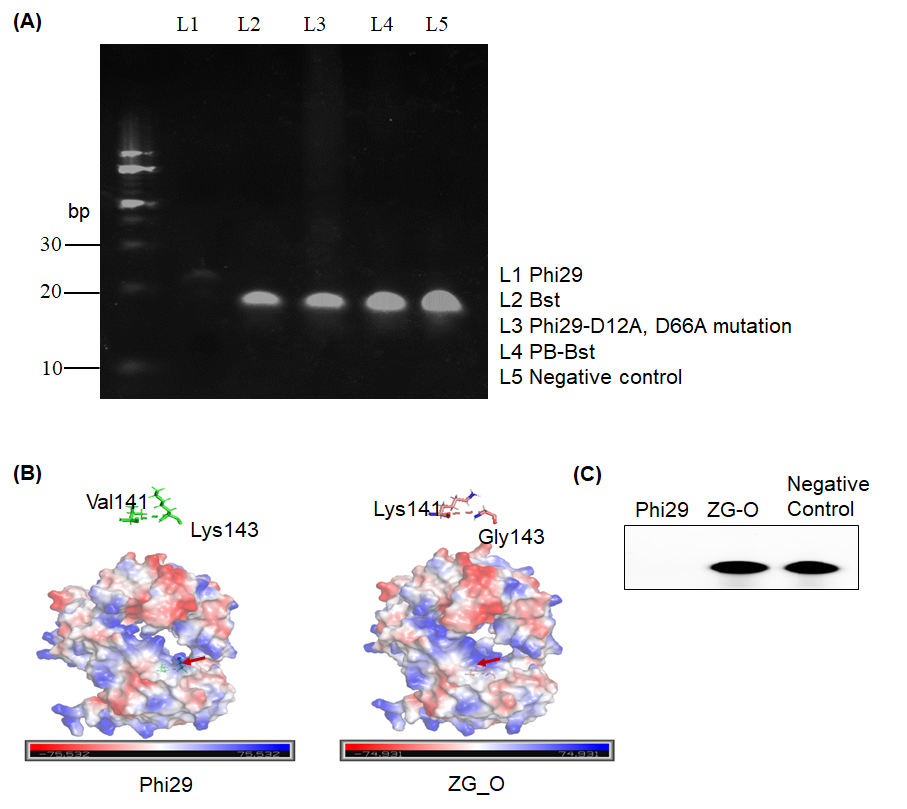

Supplement: Supplementary file 4 [file Image2.TIF]

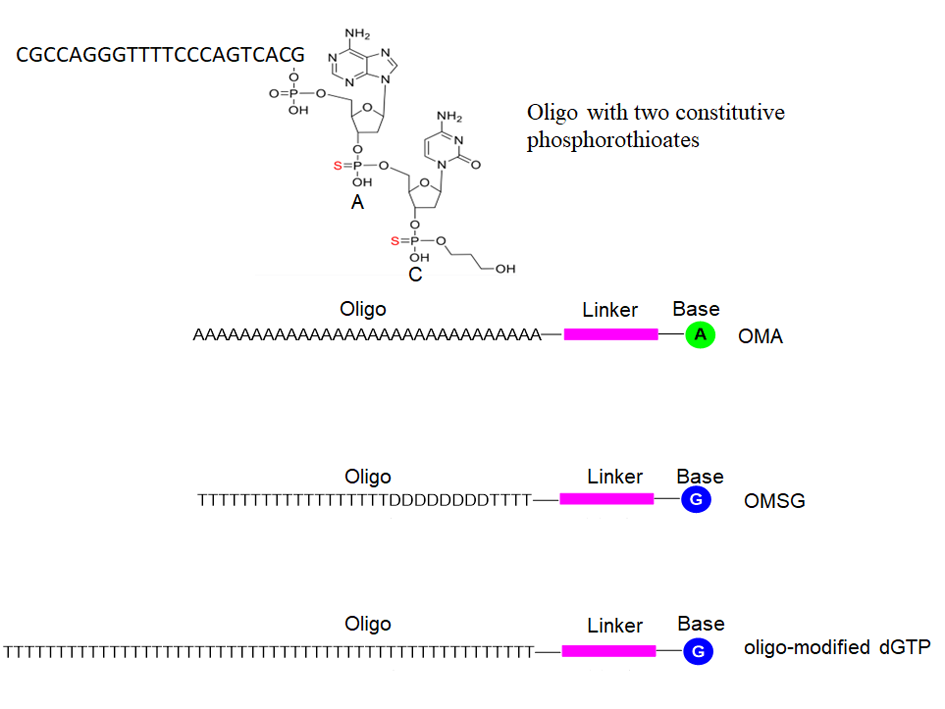

Supplement: Supplementary file 5 [file Image1.TIF]

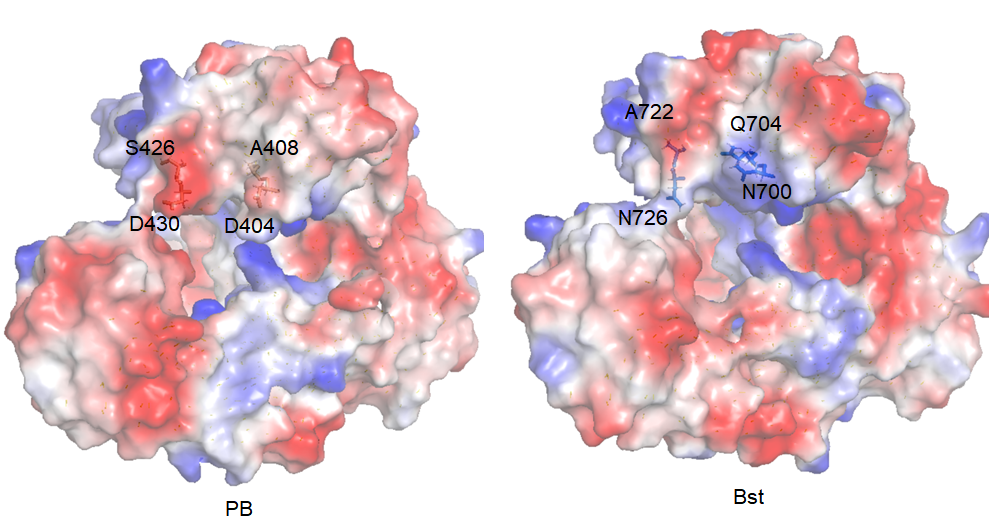

Supplement: Supplementary file 6 [file Image7.TIF]

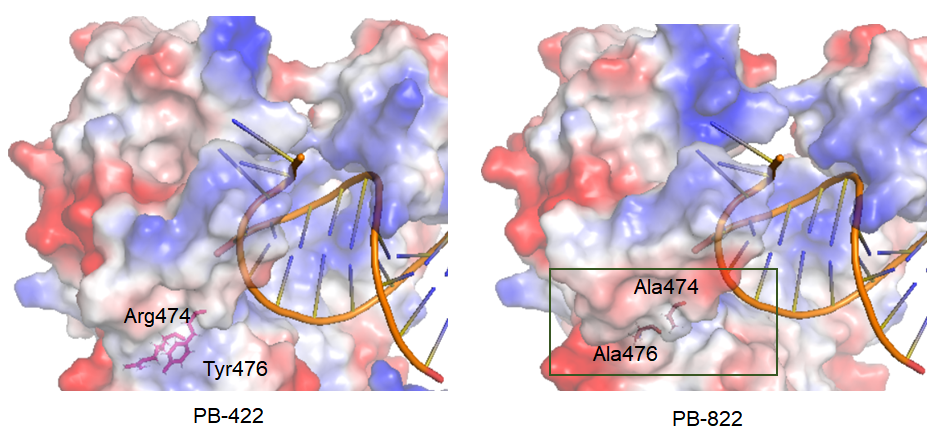

Supplement: Supplementary file 7 [file Image8.TIF]

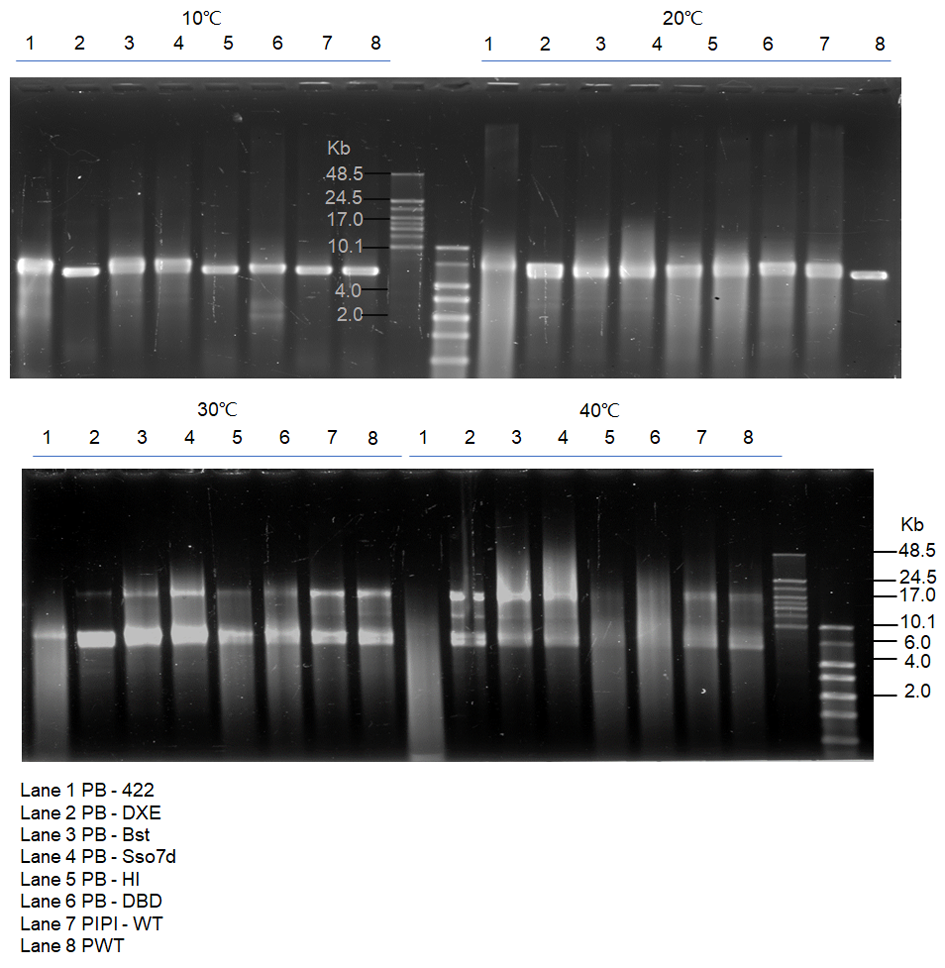

Supplement: Supplementary file 8 [file Image5.TIF]
